# Stool Sampling as a Non-invasive Method to Confirm Miliary Tuberculosis in a Patient With a History of HIV Infection

**DOI:** 10.7759/cureus.64843

**Published:** 2024-07-18

**Authors:** Kateryna Chepenko, Ulviyya Turabova, Ahmed Hassan, Tigran Kakhktsyan, Valeria Turcan

**Affiliations:** 1 Internal Medicine, Capital Health Regional Medical Center, Trenton, USA

**Keywords:** miliary lesions, sputum collection challenges, stool sampling, hiv, miliary tuberculosis, pulmonary tuberculosis

## Abstract

Tuberculosis (TB), caused by* Mycobacterium tuberculosis*, remains a major global health challenge despite medical advancements. We present here a case of a 44-year-old male with a history of HIV infection and inconsistent treatment adherence. The patient exhibited weight loss and miliary lesions on a computed tomography (CT) scan, prompting suspicion of pulmonary TB. Due to his inability to expectorate sputum, stool samples were used for the acid-fast bacilli (AFB) smear and culture. His miliary TB diagnosis was confirmed through lung CT imaging and positive AFB smears from stool samples. This case underscores the utility of stool samples in diagnosing TB when sputum production is compromised, offering a minimally invasive diagnostic approach. It also underscores the importance of collaborative healthcare approaches in managing complex cases, ensuring comprehensive care tailored to individual patient needs.

## Introduction

Tuberculosis (TB), caused by *Mycobacterium tuberculosis*, remains a significant global public health concern, contributing substantially to the worldwide TB burden [1]. The conventional method of diagnosing TB involves detecting the *M. tuberculosis *complex in sputum; this approach may present difficulties in patients unable to produce sputum, such as those with compromised immune systems or neurological impairments [2]. The diagnosis of pulmonary TB is particularly challenging also in vulnerable populations such as children, adolescents, and individuals living with HIV [3]. Nonspecific clinical symptoms, paucibacillary nature of sputum, and difficulties in collecting induced or expectorated sputum lead to frequent reliance on clinical judgment rather than definitive diagnostic confirmation, particularly among HIV-positive individuals [4]. In light of this, stool samples have emerged as a potential alternative for diagnosing pulmonary TB using molecular techniques like polymerase chain reaction (PCR).

This article was previously presented as a meeting poster "Stool Specimens: Non-invasive Alternative for Diagnosing Tuberculosis in Sputum-Inaccessible Patients" at Society of General Internal Medicine Meeting, April 13-15, 2024, San Diego, USA.

## Case presentation

**Figure 1:**
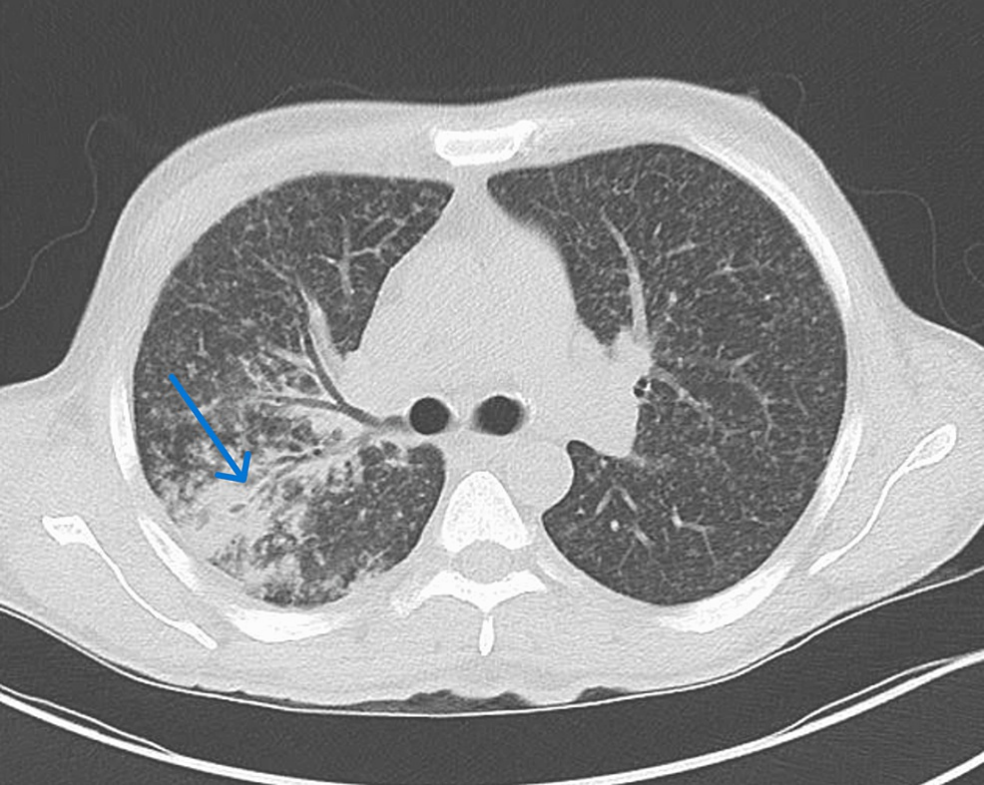
A CT scan of the lung showing irregular consolidation in the right upper lobe (blue arrow)

A 44-year-old patient with a history of HIV infection and poor treatment compliance was referred to our hospital after outpatient evaluation revealed irregular consolidation in the right upper lobe on computed tomography (CT) of the thorax (Figure 1), following a positive blood-based gamma release assay (QuantiFERON test). The patient also presented with weight loss, prompting further investigation into potential concurrent conditions. Initially, his CD4 count was 290 cells/μL in the outpatient setting and later 198 cells/μL in the hospital (Table 1). He had a history of inconsistent adherence to highly active antiretroviral therapy (HAART), leading to periods of uncontrolled viral replication. Despite this history, he had not previously experienced any AIDS-defining illnesses.

**Table 1:** Blood test results of the patient on admission

Investigation	Values	Reference range
Hemoglobin	7.8 g/dL	13.5-17.5 g/dL
Serum sodium	129 mmol/L	135-145 mmol/L
Albumin	2.4 g/dL	3.5-5.5 g/dL
CD4 count	198 cells/cubic mm	500-1500 cells/cubic mm
HIV viral load	54,000 copies/mL	0 copies/ml

**Figure 2:**
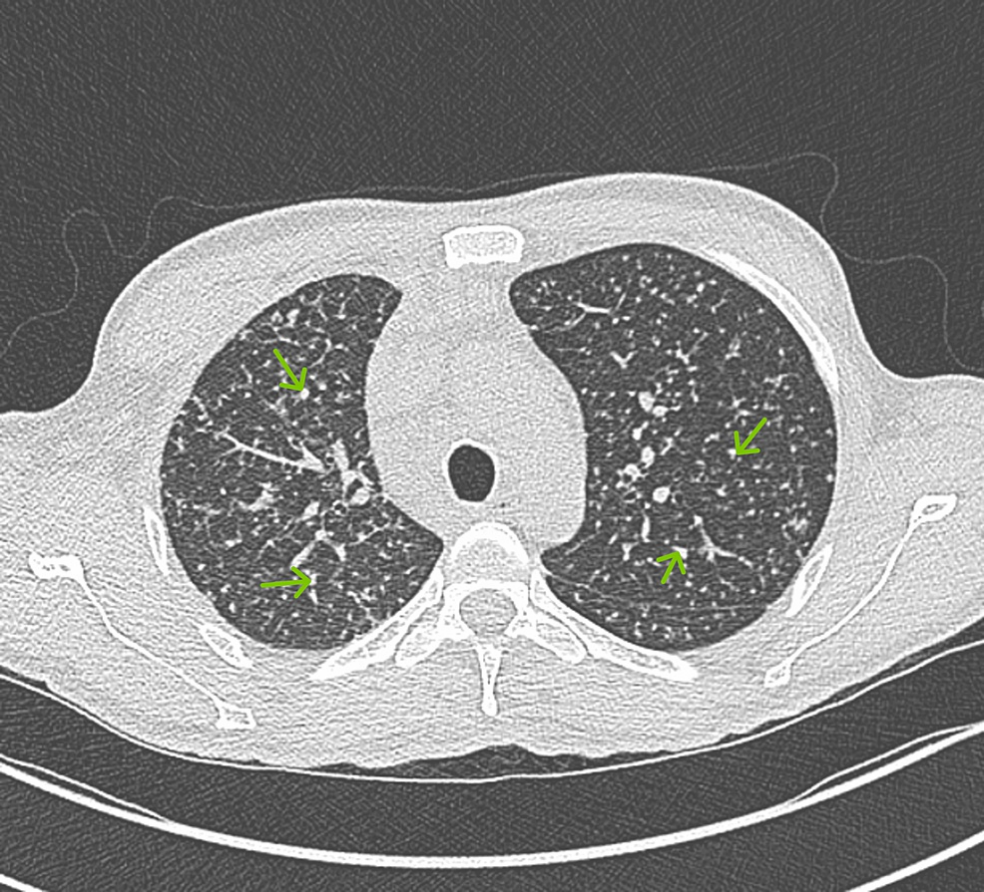
A CT scan of the lung A miliary pattern of micronodular intra-alveolar infiltrates is seen throughout the imaged portions of the lungs (green arrows). Retroperitoneal and mesenteric lymphadenopathy was found with multiple mildly enlarged lymph nodes.

Upon presentation, the patient denied respiratory symptoms, including cough or hemoptysis. A thorough physical examination was unremarkable except for mild generalized lymphadenopathy and oral thrush. Assessment in the Emergency Department revealed a miliary pattern of micronodular intra-alveolar infiltrates throughout the imaged portions of the lungs. Retroperitoneal and mesenteric lymphadenopathy was found with multiple mildly enlarged lymph nodes on contrast-enhanced CT of the thorax (Figure 2).

**Figure 3:**
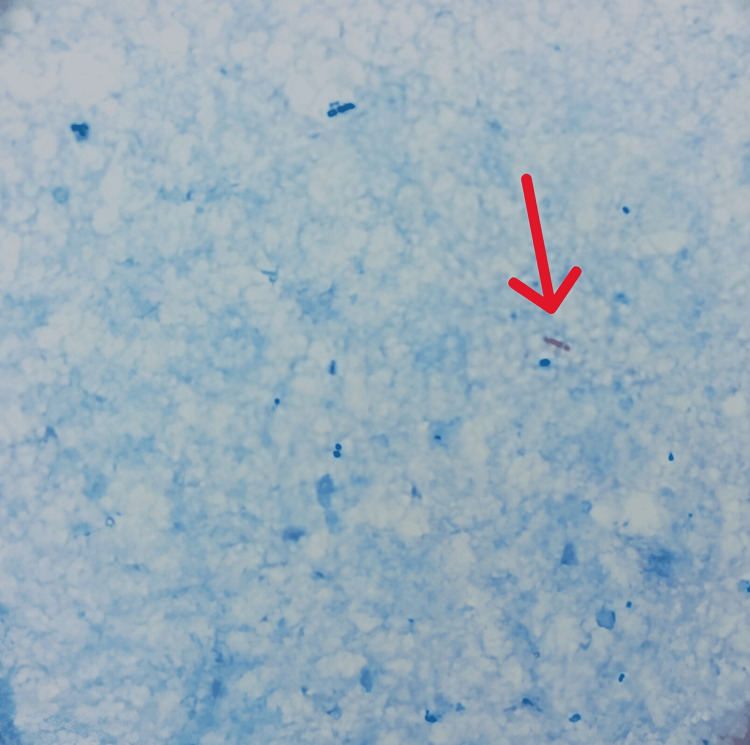
Stool smear in the Ziehl–Neelsen stain, with single acid-fast bacteria (red arrow)

The patient was admitted for inpatient care, and airborne isolation precautions were implemented. Empiric antibiotic treatment with piperacillin-tazobactam was initiated while awaiting further test results. Due to the patient's inability to expectorate sputum, sputum collection for the acid-fast bacilli (AFB) smear and culture was challenging. Consequently, the pulmonology team considered bronchoscopy to collect bronchoalveolar lavage. However, prior to invasive procedures, stool samples were obtained for AFB smears (Figure 3) as a less invasive diagnostic alternative.

Following an initial positive stool test for AFB with reflex to culture, the patient commenced complex treatment at the hospital. Subsequently, after three weeks, the culture test identified a mycobacterial infection type with negative* Mycobacterium avium complex *(MAC) but positive *Mycobacterium tuberculosis*. An antibiotic sensitivity panel was conducted, guiding the formulation of the final treatment plan, leading to the discharge of the patient.

## Discussion

While TB is often perceived as under control in the United States, it continues to pose a significant threat. According to the Centers for Disease Control and Prevention (CDC), about 13 million individuals in the United States have latent TB, with inactive bacteria. Without treatment, approximately 10% of these cases could develop into active TB, potentially leading to transmission and severe health consequences. Globally, TB remains the 13th leading cause of death and the second highest cause of infectious disease fatalities, following COVID-19.

Diagnosing pulmonary TB is particularly challenging in specific populations such as children, adolescents, and individuals with HIV. Non-specific symptoms, low bacterial counts in sputum, and difficulties in collecting sputum all contribute to diagnostic challenges, especially in HIV-positive patients [3]. The standard TB diagnosis relies on detecting *M. tuberculosis *in sputum, but this is problematic for patients who cannot produce sputum, including pediatric, immunocompromised, and neurologically impaired individuals. Early detection is crucial to reduce mortality and interrupt transmission, but the complexity and infrastructure requirements of sensitive diagnostic methods limit their accessibility and impact, particularly in resource-limited settings [4,5].

The non-invasive nature of stool sample collection offers a significant advantage over invasive procedures like nasopharyngeal aspirates, bronchoalveolar lavage, or gastric aspirates, which can be uncomfortable and risky for patients. Despite some technical challenges and relative insensitivity, stool cultures for *M. tuberculosis* have been successfully used in many developing countries, making them a practical option in resource-constrained settings. In areas with high drug-resistant TB levels, DNA from stool samples can be used for targeted next-generation sequencing, accurately predicting drug susceptibility similar to sputum phenotypic tests for first-line and second-line TB drugs.

Stool samples are particularly valuable for diagnosing TB in individuals with HIV or other immunocompromised states, who are more likely to have smear-negative and extrapulmonary TB, where sputum microscopy is less effective. In such cases, stool cultures provide an alternative diagnostic method when sputum samples are difficult to obtain [4].

Stool specimens have emerged as a promising alternative for TB detection, utilizing nucleic acid amplification techniques such as PCR to detect *M. tuberculosis *DNA. Stool specimens have been explored as an alternative method for detecting *M. tuberculosis*, leveraging the survival of bacterial DNA in swallowed sputum that passes through the gastrointestinal tract. Several studies have shown that stool samples, analyzed using nucleic acid amplification techniques such as PCR, can offer high sensitivity and accuracy, providing results within three to six hours [6-8].

For individuals with HIV, who frequently present with smear-negative and extrapulmonary TB where sputum microscopy is less effective, stool cultures offer a viable diagnostic alternative [8]. The World Health Organization (WHO) now recommends the Xpert-Ultra assay on stool samples as a primary diagnostic tool for children with presumed TB, highlighting its potential relevance in broader populations, including adults with HIV, where emerging evidence suggests enhanced diagnostic yield compared to traditional methods [3].

## Conclusions

Tuberculosis, caused by *Mycobacterium tuberculosis*, remains a formidable global health challenge, exacerbated by diagnostic complexities in vulnerable populations such as those with HIV. Traditional sputum-based diagnostics are often inadequate for patients unable to produce sputum, necessitating alternative approaches like stool sampling, which has shown promising results in detecting TB through molecular techniques like PCR. This case demonstrates the use of a cost-effective, and non-invasive technique for confirming miliary tuberculosis in adults. Typically recommended by the WHO for use in children, in our case this technique confirmed the diagnosis in a preliminary test result in three days, followed by definitive results within three weeks, including a comprehensive antibiotic sensitivity panel. This approach facilitated the selection of appropriate antibiotics, thereby enhancing the likelihood of a favorable patient outcome. As global efforts intensify to combat TB and its intersection with HIV, innovations in diagnostic methodologies, including the use of stool samples, hold significant promise in improving outcomes and reducing transmission in resource-constrained settings.
